# Diversity of Epithelial Stem Cell Types in Adult Lung

**DOI:** 10.1155/2015/728307

**Published:** 2015-02-24

**Authors:** Feng Li, Jinxi He, Jun Wei, William C. Cho, Xiaoming Liu

**Affiliations:** ^1^Center of Medical Laboratory of the General Hospital, Ningxia Medical University, Yinchuan 750004, China; ^2^Department of Thoracic Surgery of the General Hospital, Ningxia Medical University, Yinchuan 750004, China; ^3^Human Stem Cell Institute of the General Hospital, Ningxia Medical University, Yinchuan 750004, China; ^4^Department of Clinical Oncology, Queen Elizabeth Hospital, Kowloon, Hong Kong; ^5^Key Laboratory of the Ministry of Education for Conservation and Utilization of Special Biological Resources in Western China, Ningxia University, Yinchuan 750021, China

## Abstract

Lung is a complex organ lined with epithelial cells. In order to maintain its homeostasis and normal functions following injuries caused by varied extraneous and intraneous insults, such as inhaled environmental pollutants and overwhelming inflammatory responses, the respiratory epithelium normally undergoes regenerations by the proliferation and differentiation of region-specific epithelial stem/progenitor cells that resided in distinct niches along the airway tree. The importance of local epithelial stem cell niches in the specification of lung stem/progenitor cells has been recently identified. Studies using cell differentiating and lineage tracing assays, *in vitro* and/or *ex vivo* models, and genetically engineered mice have suggested that these local epithelial stem/progenitor cells within spatially distinct regions along the pulmonary tree contribute to the injury repair of epithelium adjacent to their respective niches. This paper reviews recent findings in the identification and isolation of region-specific epithelial stem/progenitor cells and local niches along the airway tree and the potential link of epithelial stem cells for the development of lung cancer.

## 1. Introduction

Adult lung is lined by surface airway epithelium. In order to meet the need of regional functionalities of the lung, the epithelia within each domain of the conducting airway are properly composed with distinct types of epithelial cells. The integrity of epithelium is essential for maintaining normal lung functions. However, the lung continually undergoes injury during the process of respiration caused by environmental insults from inhaled air; the injury repair of re-epitheliumis thus required for the preservation of epithelial integrity. In this regard, a variety of stem/progenitor cells with functional specificity are responsible for both of the injury repair and the normal turnover at steady state throughout the airway tree [1–7].

Similar to other adult tissues and organs, epithelial stem/progenitor cells in adult lung are a subset of undifferentiated cells that undergo asymmetric cell division during normal lung morphogenesis and possibly regeneration [8]. With characteristics of stem/progenitor cells, this subpopulation of cells possesses a capacity of self-renewal, proliferation, and differentiation both in a steady state and in response to injury in the physiologic domain of which they reside. According to the position within the airway tree, several epithelial cell types in the adult lung have been suggested to act as stem/progenitor cells in response to injury and exert the role in the local injury repair [1, 4, 9]. For instance, a subpopulation of distinct cell types have been demonstrated to function as progenitors or stem cells in the conducting airway of mice, such as basal cells in the proximal airway [10–16], naphthalene-resistant variant club cells within neuroepithelial bodies (NEBs) or bronchoalveolar-duct junctions (BADJ) [2, 3, 5, 6, 17–21], alveolar type II cells (AEC II) [22–24], and a subpopulation of unidentified cells in the ducts of submucosal glands (SMGs) [1, 25–28].

**Figure 1:**
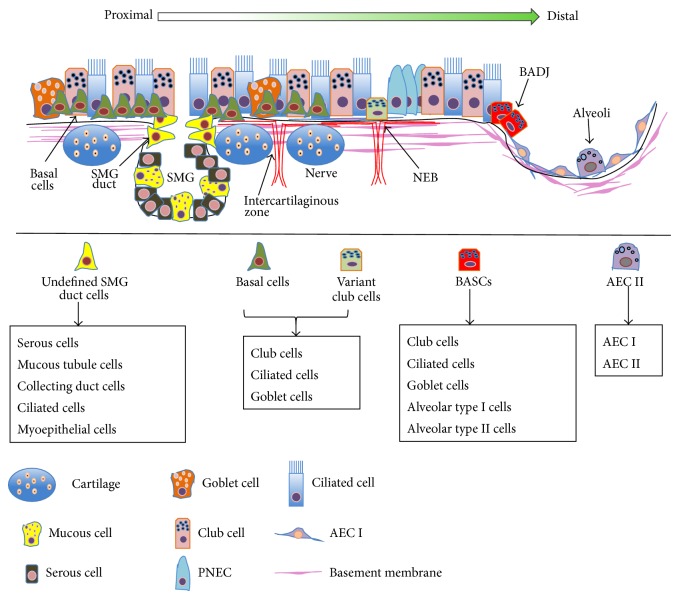
Illustration of potential stem cell niches in the adult lung. Scheme represents the regionally spatial location and distribution and differentiation of potential lung epithelial stem/progenitor cells along the airway. Distinct region-specific putative stem cell niches exist along the proximal-distal axis of the airway; they are SMG ducts in proximal trachea, basal cells of intercartilaginous zones in cartilaginous airway, NEBs in bronchioles domain, BADJ, and alveolar spaces. The potential progenitor/stem cells reside in their respective local niches, in which they maintain their stem/progenitor properties and are able to differentiate into various lung cell types. SMG: submucosal gland, NEB: neuroepithelial body, and BADJ: bronchoalveolar-duct junction.

In terms of the potential stem cell niches in lung, studies using murine models have revealed several unique regional niches for distinct epithelial stem/progenitor cell populations along the proximal-distal axis of airway, along which the epithelial stem cells reside in their specific local niches in order to maintain tissue homeostasis during injury repair and normal turnover. In this context, the coordination of local molecular and cellular events in the microenvironment of niches play pivotal roles in maintaining the balance of stem and differentiated cells for injury repair and regeneration in lung (Figure 1) [4, 9, 29–31]. Although lung epithelial stem/progenitor cells have recently been extensively reviewed [29, 32–35], this paper will focus on the diversity of epithelial cell types and potential stem/progenitor cells identified in the adult lung. In addition, the advances in our understanding of stem/progenitor cell niches and their roles in lung development, injury repair, and lung cancer will also be discussed.

## 2. Cellular Diversity in the Adult Lung

The adult lung is a highly complex organ comprised of diverse cell types, and over 40 different unique cell types with specific functions have been historically described in the lung [32, 36]. Based on the anatomical and functional features, the lung can be further divided into three epithelial domains with distinct composition of epithelial cell types, the proximal cartilaginous airway (trachea and bronchi), distal bronchioles (bronchioles, terminal bronchioles, and respiratory bronchioles), and gas exchanging airspaces (alveoli) [4].

The proximal airway is lined by pseudostratified columnar epithelial cells predominantly including basal, club, ciliated, and goblet cells and interspersed with submucosal glands (SMGs) beneath the surface airway epithelium [4, 27, 28, 37]. In distal airway (bronchioles), secretory club cells, ciliated, neuroendocrine, and goblet cells are the major cell populations. Of note, the neuroendocrine cells are found to be residing individually or as clusters in NEBs in distal airway [38]. The alveolar epithelium that leads by terminal bronchioles is lined by surfactant-producing alveolar cuboidal type II pneumocytes (AEC II) and squamous gas exchanging alveolar type I pneumocytes (AEC I) [7, 29, 32]. The major epithelial cell types and their potentials of stem cells in the adult lung are listed in the Table 1.

## 3. Potential Stems Cell Types in the Adult Lung

The epithelial stem/progenitor cells are crucial in the development, tissue homeostasis, and injury repair in lung. To date, at least a subset of basal, secretory, and mucous cells in the SMGs of the proximal airways, variant club cells in the bronchioles, bronchoalveolar stem cells (BASCs) in BADJ, and a subset of AEC II cells in alveolar space have been suggested as region-specific epithelial stem/progenitor cell populations in the adult lung of mice and humans (Table 1) [29, 32, 39].

In the cartilaginous airway (trachea and main stem bronchi), a subset of basal cells are the dominant epithelial stem cell type responsible for the homeostasis and injury repair, which possess a capacity to generate all the major epithelial cell types found in the proximal airway, including the basal, ciliated, goblet, and granular secretory cells [7, 40, 41]. Of interest, studies using murine injury model with bromodeoxyuridine (BrdU) labelling approach demonstrated that label retaining cells (LRCs) predominantly resided in the ducts of SMGs; the SMG was thus proposed as a potential stem cell niche in the proximal airway [1, 13, 37]. In addition, a recent study further demonstrated that an activation of cytokine IL6 and STAT3 signaling could promote the regeneration of ciliate cells derived from basal stem cells [16].

In order to further define the subpopulation of basal stem cells with stem cell properties, cellular markers including the cytokeratin 5 (CK5), cytokeratin 14 (CK14), and Aquaporin 3 have been employed in the identification and isolation of basal cells [11]. In this regard, Rock et al. demonstrated that the basal cells were capable of differentiating into club and ciliated cells during both steady state and injury repair of adult lung, by a lineage tracing assay using a CK5-CreERT2 mice model. In addition, they further identified that the nerve growth factor receptor (NGFR) and integrin *α*6 (ITGA6, CD49f) could be used in combination for isolation of human basal cells that have stem cell potentials [13]. Using a similar strategy, Ghosh et al. identified a CD49f^bright^/Sca-1^+^/ALDH1^+^ (aldehyde dehydrogenase 1) subset of tracheal basal cells as region-specific stem cells that could generate their own niches* in vitro* and contribute tracheal epithelial maintenance and injury repair [41]. These studies suggested that a subset of basal cells are potential stem cells that play key roles in the homeostasis and epithelial injury repair of proximal airway.

In the intralobar bronchiolar airways, a subset of variant club cells expressing Clara cell secretory protein (CCSP) but not CyP450-2F2 (CCSP^+^, CyP450-2F2^−^) show potentials of self-renewing and giving rise to club cells and ciliated cells, which meet the criteria of bronchiolar tissue-specific stem cells in adult lung [2, 5, 6, 17, 20]. Such a subset of CCSP^+^ club cells was also found at the BADJ of the distal bronchioles, at which this population of cells were responsible for repopulation of the airway epithelium after the lung injury induced by depletion of club cells using naphthalene [21]. Subsequently, Kim et al. also identified a subpopulation of club cells that coexpress CCSP and prosurfactant protein C (SPC) at the BADJ as region-specific stem cells, referred to as bronchioalveolar stem cells (BASCs) [3]. Using a naphthalene or bleomycin-induced murine lung injury repair model, they further demonstrated that these cells possessed a capacity of self-renewal and injury repair* in vivo* and an ability to differentiate into club cells and alveolar epithelial cells as determined by an* ex vivo* clonogenic assay [3]. Conversely, using a hyperoxia-induced lung injury model that the primary injury is predominantly in terminal bronchioles and alveoli, Rawlins et al. failed to observe the CCSP labelled club cells contributed injury repair of alveolar epithelium in CCSP promoter derived transgenic reporter mice, despite the fact that CCSP labelled airway epithelial cells, including the club cells and ciliated cells, were found to be repopulated following the injury as determined by an* in vivo* lineage tracing analysis [5]. These may imply the heterogeneity of variant club cell population. Indeed, studies from others also uncovered that BASCs in the distal airway might be comprised of a heterogeneous population of Sca-1^+^ cells [3, 42–44].

Other surface cell markers were also employed for the isolation and identification of BASCs. For instances, a study by Teisanu et al. showed that a subpopulation of CD45^−^/CD31^−^/CD34^−^/EpCAM^+^/Sca-1^low^/AutoFluorescence (AF)^low^ cells were variant club cells resistant to naphthalene progenitors, while the AF^high^ cells were sensitive to naphthalene [44]. Another study by Zacharek et al. also found that the BASCs could be enriched in an EpCAM^+^/Sca-1^low^/CD24^low^ cell fraction [45]. Using the same approach, Mcqualter and Bertoncello further identified an epithelial stem/progenitor cell faction EpCAM^+^/Sca-1^low^/integrin *α*6*β*4^+^/CD24^low^ which was capable of self-renewing and differentiating into the airway epithelial lineage cells, including the alveolar epithelial cells [46]. Mechanistically, a recent study by Shiomi et al. further revealed that secreted frizzled-related protein 1 (SFRP1) might be involved in the maintenance of BASCs in an undifferentiated state [47]. These studies strongly evidence that the BASCs are stem/progenitor cells that play a key role in both bronchiolar and alveolar cell injury repair and homeostasis.

In the gas exchange region, a subset of AEC II cells have long been recognized as stem/progenitor cells for AEC I cells in the adult lung [48, 49]. This notion is supported by mounting evidence from* in vitro* cell proliferation and clonogenic assays,* in vivo* epithelial injury repair, and lineage tracing analysis. These studies provided a solid evidence that a subset of AEC II cells are able to proliferate and restore the alveolar epithelium by giving rise to either new AEC II or the squamous AEC I cells following an injury; even AEC II cells have only shown their unipotency to generate AEC I cells [50, 51]. Moreover, in an attempt to identify biomarkers for AEC II stem cells, a subset of SPC^−^/integrin *α*6*β*4^+^ cells were found to sporadically localize in alveolar epithelia and were able to regenerate SPC^+^ AEC II cells in the alveoli, indicating that the expression of integrin *α*6*β*4 may be a potential biomarker for the stem/progenitor cells in alveolar epithelium [52]. Importantly, such a potency of AEC II cells as region-specific stem/progenitor cells was recently confirmed by works from Hogan's laboratory employing an* in vivo* genetically SPC-labelled lineage tracing analysis and an* in vitro* 3D culture model. In this study, the authors showed convincing evidence that AEC II cells were stem cells for maintaining the homeostasis of alveolar epithelia during the steady state and injury repair [11, 22]. Several strategies using biomarkers for identification and isolation of adult lung stem/progenitor cells are listed in Table 2.

Noteworthily, compared to the epithelial stem/progenitor cells identified in murine lungs, much less is known on stem cells in the human lung, despite the fact that several cellular markers have been employed in the identification and isolation of lung stem cells in human lung. These potential biomarkers include the Aquaporin 3 [53], c-kit (CD117) [39], NGFR and ITGA6 [11, 13, 28], Lgr6 [54], and intercellular cell adhesion molecule-1 (ICAM-1) [55]. Using fluorescence-activated cell sorting (FACS) for NGFR/ITGA [13] and HTII-280 [22], a subset of basal cells and AEC II cells have been reported to be isolated and identified as stem cells in human lung, respectively. Using similar strategies, both subsets of c-kit positive cells [39] and E-Cad/Lgr6^+^ cells [54] were also isolated and identified as putative stem cells with properties of self-renewal and differentiation* in vitro* and* in vivo *(Table 2).

## 4. Potential Niches for Epithelial Stem Cells in Adult Lung

Increasing evidence has demonstrated that distinct stem cell population resides in its specific anatomic location, “niche,” where they may work as local “emergency station” for rapidly and dynamically responding to injury and simultaneously undergoing regeneration of all necessary cell types [4, 56]. Niches are basic units of discrete microenvironments with matrix and other cells and diffusible factors such as cytokines and growth factors. A niche can functionally integrate signals to protect stem cells from depletion and governor stem cell self-renewal, proliferation, and differentiation. For instance, the fibroblast growth factor 10 (FGF10) has recently been suggested to play a central role in the communication between mesenchymal tissues and epithelial cells in lung stem cell niches [35]. The interaction between the stem cells and their niche environments creates a dynamic system that is critical for maintaining the potency of stem cells and promoting appropriate cell fate and migration decisions [1, 4, 17].

Using murine injury model and lineage tracing analysis, at least six putative stem cell niches have been proposed in the adult lung; they are the duct of SMG, intercartilaginous zone surface basal cells of tracheobronchial airways [1, 4, 11, 13, 26], neuroepithelial bodies (NEBs) of the bronchi and bronchioles [2, 17, 26, 50], bronchoalveolar-duct junction (BADJ) of the bronchiolar epithelium [3, 21, 42–45], and the alveolar epithelium of the terminals of lung (Figure 1) [22, 51, 57].

The SMG in cartilaginous airway is one of the best currently characterized stem cell niches in the lung, where a subpopulation of undefined duct cells have recently been identified as stem/progenitor cells with capacity to repopulate both of glandular cells and airway surface epithelial cells following a severe injury [1, 4, 27, 28, 37]. Functionally, the SMGs play key roles in maintaining normal lung function and innate immunity of lung by secreting antibacterial factors, mucous, and fluid into the airway lumen [58]. They are also thought to play an important role in the pathogenesis of a number of hypersecretory lung diseases, such as cystic fibrosis (CF), chronic bronchitis, and asthma, where the severe hypertrophy and hyperplasia of submucosal glands are characteristic of the progressing diseases [59, 60].

Using a combined approach of tracheal xenograft with* ex vivo* genetically retroviral labelling, Engelhardt and colleagues evaluated progenitor/progeny relationships and the existence of a stem cell compartment in the adult human proximal airway [37]. Epithelial reconstitution using this approach uncovered the existence of transgenetically marked SMGs that infrequently developed in these xenograft airways. Of great interest, transgene-expressing glands were always associated with pluripotent transgene-expressing clones in the surface airway epithelium, suggesting a subset of progenitors with pluripotent capacity for surface airway epithelial differentiation and for SMGs development [37]. Consistent with the finding of the capacity of airway stem cells to form SMGs, a subset of LRC cells from glands or gland ducts could reconstitute the tracheal epithelium following the SO_2_ or naphthalene induced lung injury, suggesting that subsets of cells located in the SMGs of conducting airway behaved in a manner consistent with tissue-specific stem/progenitor cells. These cells were permanently established in the airway and were capable of differentiation for the regeneration of airway epithelia upon an injury, although the phenotype of LRCs in the SMGs has not yet been characterized [1]. Such notion was supported by recent findings using both of murine hypoxic-ischemic injury model and syngeneic heterotopic tracheal transplant model [27, 42]. In these studies, Hegab et al. found that the SMG duct cells were distinct stem/progenitor cell population shared similarities with basal cells, which could survive during the injury and were capable of self-renewal and of reconstitution of SMGs and surface epithelium* in vivo* [27, 42]. Herein, these studies provide conceivable evidences that the SMGs may serve as the glandular stem cell niches to protect stem cells from the injury and airway epithelial repairing after the injury, although the exact cell type of duct cells has not been defined yet.

Apart from the SMG ducts, the surface basal cells in intercartilaginous zone of tracheobronchial airways were also to be suggested as stem cell niches in proximal airway [1, 4, 11, 13, 26]. Using murine injury model, Borthwick et al. found that a subset of cytokeratin-5 (CK5) and -14 (CK14) positive cells located at intercartilaginous zone was BrdU LRCs [1]. Such a subpopulation of basal cells with capacity of self-renewal and differentiation were also identified by others using clonogenic assay and cell lineage tracing analysis [12–15, 40]. Of note, Rock et al. recently identified a subset of p63^+^/NGFR^+^/CK5^+^ basal cells that were able to self-renew and generate luminal daughter cells as defined using a 3D tracheosphere assay in a Matrigel culture model [13]. Furthermore, they further demonstrated that CK5-basal cells could give rise to ciliated cells in the tracheobronchial airways during both the steady-state and injury repair by lineage tracing analysis using a murine model of CK5–CreER transgenic mouse [7, 11]. Together with the fact of abundant blood vessel and nerves in the intercartilaginous zones and NEBs of airway, these studies suggest that the intercartilaginous zone and NEBs are locations for stem cell niches.

In the bronchiolar airway, the calcitonin gene-related peptide (CGRP) marked NEBs have been proposed as a niche for lung stem/progenitor cells responsible for the homeostasis of bronchiolar epithelium during normal turnover and injury repair [2, 61]. In this regard, at least two distinct cell types have been identified in the NEBs, the CCSP^+^/CyP450^−^ variant club cells, and the pulmonary neuroendocrine cells (PNECs) expressing CGRP [18, 62]. Several studies using lineage tracing analysis in murine models revealed that both of CGRP-expressing PNECs [63] and a subset of variant club cells exhibited a capacity to self-renew and to differentiate into club and ciliated cells following the naphthalene injury [2, 5, 17, 20]. In addition to the identification of a subpopulation of the naphthalene resistant CCSP^+^/CyP450^−^ variant club cells as putative stem/progenitor cells in the distal airway [2, 5, 18, 62], Xing et al. [20] recently identified a distinct subset of CCSP^low^/CyP450^−^/Scgb3a2^+^ expressing club cells residing in the NEBs in which Notch signal and transcription factor TTF1 (Nkx2.1) played a crucial role in the induction of secretory cell fate determination in developing murine airways [17]. These studies support that the NEB microenvironment may be a stem cell niche for these club precursors. In addition, the importance of Notch signal in club cells was also found in adult lung, in which the Notch1 was required for repopulation of club cells during the injury repair of the airway epithelium. It is worthy to note that CGRP-PNECs previously demonstrated an inability to regenerate CCSP-club cells when all club cells (including the variant club cells) were ablated by administration of ganciclovir to transgenic mice harboring CCSP promoter derived thymidine kinase (TK); that is, PNECs were insufficient for reepithelialization in mouse airway [2]. In support of this finding, Song et al. created a genetically modified murine model with a Cre recombinase/CreER fusion gene introduced into the CGRP locus. Lineage tracing analysis using this model revealed that PNECs could contribute to club cells and ciliated cells. However, consistent with the finding by Hong et al. [2], an ablation of PNECs had no overt impact on club cell regeneration [63]. These studies suggest that the biological function of CGRP-PNECs in lung homeostasis and injury/repair needs to be further characterized.

In the terminal bronchioles, mounting evidence has suggested that the BADJ is the niche for BASCs that are capable of regeneration of both bronchiolar and alveolar epithelial cells in response to injury [3, 44, 45, 64–67]. Despite the fact that* in vitro* studies revealed the capacity of BASCs to differentiate into club, AEC II, and AEC I cells [3, 21],* in vivo* lineage tracing study using CCSP-CreER murine model showed an inability of CCSP positive cells to contribute alveolar injury repair following a naphthalene or hyperoxic acute lung injury [68]. Nevertheless, the CCSP-labelled AEC I and AEC II cells could be regenerated in the alveolar injury model induced by either influenza viral infection or bleomycin exposure [19, 65, 66]. These studies indicate that the potential of BASCs in alveolar injury repair may be injury-specific and dependent on specific microenvironmental factors at BADJ, a niche for BASCs in distal airway.

In the terminal end of respiratory tree, AEC I and AEC II cells, capillaries, and lung resident mesenchymal cells (lrMSCs) comprise gas exchange area, the alveolar ducts, and alveolar sacs. The AEC II cells have been histologically suggested as the stem/progenitor cells responsible for regeneration of AEC I and AEC II cells after an alveolar injury [21, 22, 52]. However, in a recent study using an SPC-inducible CreER murine model and a bleomycin injury approach, Chapman et al. found that the majority of AEC II cells in the fibrotic areas were not repopulated from preexisting SPC^+^-AEC II cells, suggesting that SPC expressing cells were not the major contributors for alveolar repair following the bleomycin-induced parenchymal injury [52]. Instead, they identified a subset of previously unrecognized AECs expressing laminin receptor integrin *α*6*β*4, but not the AEC II cell marker SPC, was progenitor cell type. This subset of AECs was able to proliferate and differentiate into many epithelial cell types, including the CCSP-expressing cells and SPC^+^-AEC II cells in an* ex vivo* kidney capsule model [52]. Conversely, a more recent study by Barkauskas et al. further revealed that the SPC positive AEC II cells had ability to self-renew and differentiate over about a year, as well as rapid clonogenesis of individual survivor cells when the majority of AEC II cells were specifically ablated* in vivo*, supporting the existence of alveolar stem cell population [22]. Furthermore,* in vitro* differentiation analysis using a 3D culture model showed that an individual AEC II cell could give rise to self-renewing “alveolospheres” comprised of both AEC I and AEC II cells. Noteworthily, a coculture of the AEC II and primary PDGFR*α* ^+^ lung stromal cells showed most readily growth and differentiation of the alveolospheres. The primary PDGFR*α* ^+^ lung stromal cells include a population of lipofibroblasts (lrMSCs) that reside close to AEC II cells which may play an important role in the epithelial-mesenchymal interaction-generated alveolar lung development [69] and serve a stem cell niche in the lung [22].

## 5. Stem Cell Niches and Lung Cancer Stem Cells

**Figure 2:**
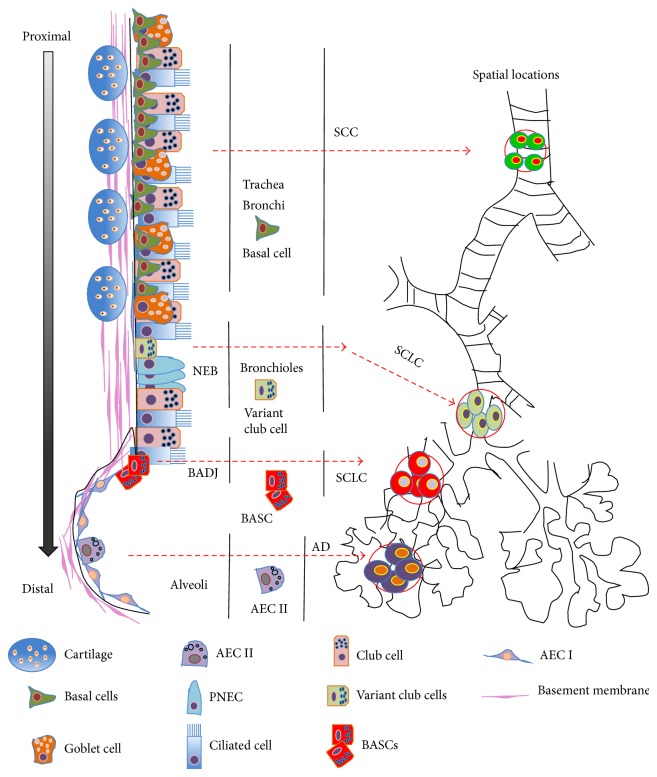
Illustration of epithelial stem cells and lung cancers along airway tree. Scheme represents the special localization of regional-specific epithelial stem cells and the respective potential for initiations of distinct lung cancers. NEB: neuroepithelial body; BADJ: bronchoalveolar-duct junction.

Lung cancer is a heterogeneous and complex disease in terms of its diversity of phenotypes and anatomical sites of origins in airways. It thus can be subdivided into two major groups: small cell lung cancer (SCLC) and non-small cell lung cancers (NSCLC). The SCLC is characterized by neuroendocrine cell morphology and accounts for ~15% of lung malignancies; however the NSCLC accounts for the rest ~85% of lung malignances and can be further subdivided into three distinct histological subtypes, which are squamous cells carcinoma (SCC), adenocarcinoma, and large cell carcinoma [70]. Such heterogeneity and the initiation, progression, chemoresistance, recurrence, and metastasis of lung cancer have been ascribed to lung cancer stem cells (CSCs) uniquely endowed with capacity self-renewal and proliferation [71–73]. To date, despite the fact that there is no universal lung CSC marker that has been defined, several tumor markers including the aldehyde dehydrogenase (ALDH), CD133, and CD44 were used for lung CSC isolation, by which the heterogeneity and cellular plasticity of lung CSCs were found [72, 74, 75]. Although the CSCs in lung cancer are less characterized as compared with the CSCs in other tumors, the increased understanding in the properties of region-specific airway epithelial stem/progenitor cell populations has led to hypothesis that the anatomical locations of these stem cell populations are responsible for the regional diversity of origin and type of lung cancers. In this regard, a distinct phenotype of lung cancer shares the characteristics with the corresponding regional stem/progenitor cell population responsible for the injury repair of this area [3, 68, 71, 76–78]. This hypothesis is supported in part by findings from animal models of induced lung cancers, where lung cancers originated from resident stem cells, and the most originating sites of different types of lung cancer are correlated with distinct airway stem cell niches (Figure 2) [24, 33, 34, 79, 80]. In this context, the adenocarcinomas are characterized with expression of CK14, transcription factor Nkx2.1 (TTF1), CCSP, and SPC and arose from the BADJ in murine model, suggesting that club cells or AEC II cells stem/progenitor cells may be the initiating cells for adenocarcinomas in distal lung [3, 70, 71, 78, 81, 82]. Lung SCCs are characterized by squamous differentiation with basal cell phenotype persistent expressions of CK5, p63, and Sox2, which are often found at the ducts of SMGs or at intracartilaginous boundaries in the trachea and upper airways where the most abundant basal cells are located, indicative of basal cell origins of SCC [70, 76, 81, 83]. Similarly, SCLCs are predominantly found in the intermediate airways and characterized with expression of a range of neuroendocrine markers, including the CGRP within the NEBs, suggesting that SCLCs may originate from the NEBs. This hypothesis is further supported by a finding in Rb1- and p53-deficient mice, in which SCLCs are most frequently developed from the PNECs within the NEBs. This was in part attributed to Rb1- and p53-deficient in the club cells, AEC II cells, and PNECs, as a function of Rb1 gene is negative regulation of neuroendocrine differentiation [76, 84]. These studies clearly suggest that stem cell specific microenvironments (niches) may play a key role in supporting CSCs self-renewal and proliferation, subsequently promoting the initiation, malignance, chemoresistance, and recurrence in lung cancer [24, 85].

## 6. Prospective 

Using* in vitro* and* in vivo* clonogenic assay and lineage tracing analysis, a picture of coordination between the region-specific epithelial stem cells and their distinct niches has displayed. A balance of the region-specific stem cells and their distinct residential niches is pivotal for maintaining normal functions and homeostasis of lung along the airway during both the steady state and injury repair. In this context, stem cell niches located at different regions of the lung play crucial roles in protecting stem cell from depletion, and they govern the specificity of regional stem cells for normal lung turnover and injury repair. An abnormal niche activity may result in lung diseases, including lung cancer. Therefore, defining the intrinsic and extrinsic cues between lung stem cells and their unique regional niches may ultimately provide novel insights into the progresses underpinning the pathogenesis of lung diseases involving abnormalities injury repair and lung CSCs.

Although the markers and locations of several epithelial stem/progenitor cells of airway have been interrogated in great detail, such as the BASCs at BADJ and the basal cells in proximal airways, a specific marker for the identification and isolation of lung stem cells has not been defined yet. More phenotypic markers are thus needed for the unambiguous identification and isolation of lung stem cells. In addition, other unidentified subpopulations of stem/progenitor cells may be very important which has been isolated. On the other hand, an overlap immunophenotypic profile of BASCs identified from independent groups implies that there is heterogeneity among the stem cell populations. This may suggest that a variably enriched population of stem/progenitor cells could be isolated from different locations. Hence, defining better specific surface markers is necessary for the isolation of a homogenous population of lung stem cells. Equally important is the fact that an analysis at a single cell level may also enable us to interrogate a more detailed and precise lineage relationships of stem cells.

Apart from the identification of the locations (niches) of lung stem cells, the molecular signaling network of niches involved in the control of stem cell fates is mostly unclear. Therefore, the understanding of the molecular structure and composition, as well as the signaling pathways of distinct niches, will allow us to better understand behaviors of different region-specific stem cells in lung.

## Figures and Tables

**Table 1:** Major epithelial cell types in adult murine lung.

Cell type	Cellular marker	Progenitor/Stem cells	Lineage cell type(s)	Candidate niches	Reference(s)
Basal cells	Cytokeratin 5, cytokeratin 14	Yes	Basal, secretory, mucous, ciliated cells, PNEC	SMGs, NEBs, intercartilaginous zone	[13–15, 40]
Club cells	CCSP, CyP450 2F2	Yes	Club, mucous, ciliated, AEC I, AEC II cells, PNEC	NEBs, BADJ	[2, 5, 18, 86]
Ciliated cells	FoxJ1, Tubulin IV			SMGs	[61, 87, 88]
Mucous cells	Mucin 5AC, Mucin 5B	Yes	Basal, mucous, ciliated cells	SMGs	[12, 37, 89]
AEC I cells	Aquaporin 5	No	AEC I		[4, 22, 89]
A EC II cells	SPA, SPB, SPC, LysoTracker DND-26, Aquaporin 1	Yes	AEC I, AEC II, club cells, PNEC	Alveoli space	[4, 22, 23, 33, 50, 90]
PNEC	CGRP	No	Club cells	NEB	[2, 63, 91]

**Table 2:** Subsets of epithelial cell types identified as potential stem/progenitor cells in adult lung.

Cell type/markers	Lineage cell types	Assay	Reference(s)
Keratin 5^+^/basal cells	Mouse airway epithelial cells	*In vitro *	[11, 13, 26, 40]
H33342^+^/Sca^+/−^/CD45^+/−^/HNF3n^+^	Mouse airway epithelial cells	*In vitro *	[5]
CCSP^+^ variant club cells	Mouse airway epithelial cells	*In vivo *	[92]
Sca-1^+^/CD45^−^/CD31^−^	Mouse airway epithelial cells	*In vivo *	[42]
Sca-1^+^/CD45^−^/CD31^−^/CD34^+^	Mouse airway epithelial cells	*In vivo, in vitro *	[93]
Sca-1^+^/CD49f^high^/ALDH1^+^	Mouse airway epithelial cells	*In vitro, in vivo *	[41]
Sca-1^low^/AF^low^/CD45^−^/CD31^−^/CD34^−^	Mouse bronchiolar epithelial cells	*In vivo *	[44, 94]
Sca-1^+^/CD34^+^/CCSP^+^/SPC^+^	Mouse bronchiolar and alveolar epithelial cells	*In vivo *	[3]
CD45^−^/CD31^−^/EpCAM^hi^/CD49f^+^/CD104^+^/CD24^low^	Mouse lung epithelial cells	*In vitro *	[67, 94]
EpCAM^−^/CD45^−^/NGFR^+^/ICAM1^+^	Human nasal epithelial cells	*In vitro *	[55]
NGFR^+^/ITGA6^+^	Human tracheal basal epithelial cells and SMG duct cells	*In vitro *	[11, 13, 28]
CD151^+^/tissue factor^+^	Human airway epithelial cells	*In vitro *	[14]
Human SP/CD45^−^	Human airway epithelial cells	*In vitro *	[95]
CD45^−^/CD31^−^/EpCAM^+^/Sca-1^low^/CD24^low^	Bronchiolar and alveolar epithelial cells, BASC	*In vitro *	[45]
c-kit (CD117)	Human lung epithelial cells	*In vivo *	[39]
Ecad/Lgr6^+^	Human lung epithelial cells	*In vitro, in vivo *	[54]

TF: tissue factor; ICAM: intercellular adhesion molecule 1.
